# Cell therapy for type 1 diabetes

**DOI:** 10.1093/qjmed/hcu025

**Published:** 2014-03-07

**Authors:** K.R. Muir, M.J. Lima, H.M. Docherty, K. Docherty

**Affiliations:** From the School of Medical Sciences, University of Aberdeen, Institute of Medical Sciences, Aberdeen AB25 2ZD, UK

## Abstract

Cell therapy in the form of human islet transplantation has been a successful form of treatment for patients with type 1 diabetes for over 10 years, but is significantly limited by lack of suitable donor material. A replenishable supply of insulin-producing cells has the potential to address this problem; however to date success has been limited to a few preclinical studies. Two of the most promising strategies include differentiation of embryonic stem cells and induced pluripotent stem cells towards insulin-producing cells and transdifferentiation of acinar or other closely related cell types towards β-cells. Here, we discuss recent progress and challenges that need to be overcome in taking cell therapy to the clinic.

## Introduction

It is just over 90 years since J.J.R. Macleod and his team at the University of Toronto discovered insulin.^1^ The first patient, Leonard Thompson, at the time of treatment was on a starvation diet that was intended to extend his life for a few years. He was injected with a crude extract of bovine pancreas in January 1922 with an almost immediate effect on his glycosuria, blood glucose levels and general well-being. From that moment onward diabetes was no longer a fatal disease. Since then, injection of exogenous insulin has been in the vanguard in the battle to control the disease; the aim being to simulate the normal pattern of insulin secretion as closely as possible. This has been best achieved by basal-bolus therapy using multiple daily injections or continuous subcutaneous insulin infusion pumps. There have been many major breakthroughs since 1922, but none more important than the cloning and sequencing of the insulin gene in 1980,^2^ which brought about the introduction of unlimited supplies of bacterially expressed human insulin and the technology to modify the structure of the protein, such that there are now at least six rapid-acting or long-acting analogues. Combined with advances in glucose monitoring, these modified insulins have allowed patients to control their blood glucose levels within relatively narrow limits. Achieving tight glycaemic control with current medical therapy is, however, something of a double-edged sword. It has led to a fall in the microvascular complications namely retinopathy, nephropathy and neuropathy, reducing patient morbidity, but at a consequence of increased rates of disabling hypoglycaemia. With diabetes now reaching epidemic proportions affecting around 6% of the adult population in the UK, and type 1 diabetes (T1D) accounting for 5–10% of all cases, new therapeutic strategies are clearly required to reduce this colossal health and economic burden.

Along with improvements in insulin therapy there have been huge advances in our understanding of the disease. T1D is an autoimmune disorder in which activated CD4+ and CD8+ T lymphocytes infiltrate the islets of Langerhans and selectively destroy the β-cells. Diagnosis is typically during childhood but can occur at any age, by which time 70–80% of the β-cell mass is already lost through apoptosis. The cure for T1D is likely to come from immune interventions directed at preventing the disease prior to the establishment of autoimmunity.^3^ For those people with established T1D progress has been made in identifying targets for vaccines, but there have been major challenges in establishing realistic end-points for immunotherapeutic trials.^4^ In the meantime improved insulin therapy, with emphasis on closed loop delivery systems or islet transplantation, is generally accepted as the best way forward. A comparison of continuous glucose monitoring data from patients on closed loop delivery systems and those that have undergone islet transplants indicates that current closed loop delivery systems cannot get close to matching the control that can be achieved by islet transplantation.

## Current cell therapy

Islet transplantation mainly in the context of syngeneic transplantation following removal of the pancreas in patients with pancreatitis has been around since the early 1990s.^5^ The success rate for syngeneic islet transplants has been relatively good, but allogeneic transplantation of donor islets for the treatment of T1D was plagued from the outset with poor success rates; 8% graft function after 1 year. This changed with the introduction of the Edmonton Protocol in 2000, which placed emphasis on transplanting a sufficiently large number of islets (typically 2–3 donors), minimizing the cold ischemia time and implementing an immunosuppressive regimen without corticosteroids.^6^ With further improvements in immunosuppression, clinical islet transplantation has progressed considerably such that by the end of 2013 over 750 patients with T1D have received transplants. The 1-year success rates are high particularly with regards to reduction in severe hypoglycaemia, although there are still concerns about graft failure with time.^5^ If advances could be made in addressing the allo- and auto-immune islet attack in the recipient, then the procedure could be made more widely available for patients with T1D. However, before this can be envisaged there is a requirement for a replenishable supply of islets that is not dependent on cadaveric tissue. This brief review will describe how progress in stem cell research has been channelled towards this goal with emphasis on our own experience in the differentiation of pluripotent cells and the reprogramming of adult tissue.

## Future cell therapy

### Differentiation of pluripotent cells towards β-cells

Pluripotent cells can be obtained from the inner cell mass of pre-implantation embryos [embryonic stem cells (ESCs)] or from the reprogramming of adult tissues to generate induced pluripotent stem cells (iPSCs). Mouse ESCs have been around since the early 1980s.^7^^,^^8^ Human ESCs differ significantly from mouse ESCs, probably because they arise from a later stage in the developing blastocoel that is more closely related to the mouse epiblast than the inner cell mass of the fertilized egg.^9^ They require very different culture conditions and this in part explains the lengthy time period between the derivation of the first mouse and human ESCs in 1998.^10^ Since then around 800 human ESC lines have been generated, although they vary markedly in their quality and only a very small proportion of these are in common use. iPSCs were first generated from mouse embryonic and adult fibroblasts following transduction with retroviruses containing four transcription factors, Oct4, Sox2, Klf4 and c-Myc that are normally expressed in ESCs.^11^ It was very quickly established that iPSCs could be generated from a variety of adult human cell types.^12^ In common with ESCs iPSCs can theoretically be induced to differentiate into any of the 200 or so cell types in the body, and thus like ESCs have the potential to provide an essentially unlimited supply of specific cell types for basic research and transplantation therapies for disease.

In terms of providing a replenishable supply of islets for transplantation, it is likely that a small number of pluripotent lines will be employed. The reason for this is that each cell line would need to be generated, maintained, and differentiated under clinical GMP-grade conditions and expanded to meet demand. A healthy pancreas contains about 1 million islets and an average-sized islet contains a few hundred β-cells. This suggests that something like 1 billion differentiated cells would be required for each transplant. At clinical presentation most people with T1D have some (∼10%) residual β-cell function, suggesting that the bar could be lowered. Nonetheless, this would still require cell culture on an industrial scale, which could only be carried out by commercial companies that are skilled in handling cell factories, as presently used for the production of therapeutic monoclonal antibodies. At present, there are a handful of ESC lines that would fit these requirements. However, in the future, there is a case for generating a bank of pluripotent cells selected on the basis of their growth, differentiation capabilities and HLA-type. Islet transplant recipients are not usually HLA-matched with donors. However, despite being immunosuppressed, there is evidence that HLA-matching would provide benefits from at least partial HLA-matching.^13^ The ideal pluripotent stem cell bank should therefore be sufficiently large to cover the range of MHC compatibility within regional or racial genetic backgrounds.^14^ For ESCs this would involve screening an enormous range of lines from embryos harvested from young women, a procedure which is not without risk to the donor, and which raises considerable ethical issues. iPSCs on the other hand can be generated from easily accessible adult tissues with little or no risk to the donor. iPSCs generated from specific patients could have the potential to provide insights into disease mechanisms; however, in terms of cell therapy a limited number of MHC-specific iPSC lines could be generated from tens of thousands of donors and further selected on the basis of their growth characteristics and ability to differentiate down endoderm, mesoderm or ectodermal lineages.^15^

Although ESCs and iPSCs share marked similarities, there are subtle genomic differences within the two populations and between these and non-pluripotent cells.^16^ The origin of the genomic alterations in iPSCs can be attributed to their pre-existence in the parental somatic cells or their acquisition during reprogramming. Culture adaptations can also contribute to these defects for both ESCs and iPSCs.^17^ However, the functional consequences of these genomic aberrations, which for the most part are epigenetic, i.e. do not involve changes in DNA sequence, are not known. Epigenetic changes may even have a positive effect on the differentiation potential of iPSCs. At this stage, the advantages that iPSCs provide in terms of generating an autologous bank of HLA-typed pluripotent cells likely outweigh any unknown consequence that epigenetic abnormalities (some of which are shared with ESCs) might have on their clinical utility.

**Figure 1.**
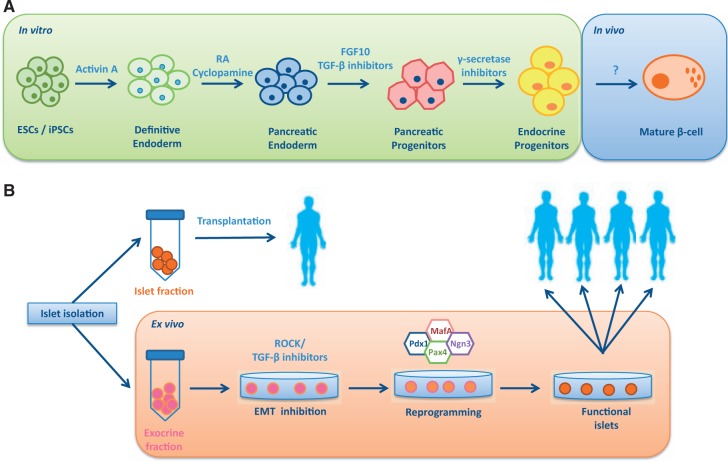
Strategies for generating a replenishable supply of beta cells. (**A**) Schematic representation of the stepwise differentiation of pluripotent cells towards β-cells *in vitro*. The final maturation stages of beta cell development remain challenging to replicate under *in vitro* conditions. (**B**) Procedure for reprogramming the exocrine tissue resultant from the islet isolation procedure towards functional beta cells. After being placed in culture, the exocrine phenotype is maintained by inhibiting EMT. Reprogramming towards functional beta cells is subsequently achieved by overexpression of pancreatic transcription factors and growth factors.

Considerable progress has been made in developing protocols for the efficient differentiation of pluripotent cells towards functional islets (Figure 1A). The strategy is influenced by advances in understanding the mechanism that control pancreatic development in the mouse.^18^ The first step (2 days, D0–D2) is to induce formation of definite endoderm by including high concentrations (100 ng/ml) of activin A in the culture media. Activin A mimics the effects of nodal signalling in the early embryo. The next stage (D2–D4) involves specification of the pancreas, which is achieved by adding retinoic acid and inhibiting endogenous sonic hedgehog signalling with cyclopamine. Formation of the pancreatic cell types is then achieved by adding fibroblast growth factor (FGF) and inhibiting the actions of activin A (D4–D6), which at this stage would push the cells towards liver lineages.^19^ Inhibiting Delta/Notch signalling (D7–D9), by use of a γ-secretase inhibitor, results in an enriched population of endocrine progenitors. To date, it has not been possible to differentiate these progenitors further into fully functional β-cells;^20^ however when placed under the kidney capsule or epididymal fat pad of immunocompromised mice, the progenitors, after 12 weeks or so, secrete human C-peptide in a manner that responds to a glucose tolerance test and can rescue hyperglycaemia if the mice are subsequently treated with streptozotocin, which kills mouse but not human β-cells.^19–21^ These findings, including variations of this blueprint, have been reproduced in a number of laboratories, and at this stage proposals are being drafted to take islet progenitors into clinical trials. The plan would be to microencapsulate the progenitor cells in devices similar to those produced by Theracyte™,^22^^,^^23^ where maturation to functional β-cells would take place. Encapsulation is particularly important for safety reasons so that at the first sign of any mal-function, the graft can be removed. The ideal microencapsulation device would also act as an immunobarrier. It is important to note that newly diagnosed T1D patients with residual C-peptide at the lower end of the normal range (∼73 pM) exhibit a good response to a mixed meal tolerance test. So even producing small amounts of insulin would have a beneficial effect not only in terms of glycaemic control but also with respect to reducing fear of hypoglycaemia.

### Reprogramming adult cell types towards β-cells

The idea that one differentiated human cell type could be converted into another would have been met with ridicule not so many years ago.^24^ There was evidence that some cells could transdifferentiate during embryogenesis or under highly specific circumstances, but this was not a property generally associated with differentiated tissues. However, the ground-breaking work of Takahashi and Yamanaka, showing that essentially any tissue could be reprogrammed towards pluripotent cells, i.e. iPSCs, revolutionized the concept of tissue plasticity.^11^ Prior to this there were data around showing that exocrine pancreatic cells could be induced to transdifferentiate into β-like cells,^25^ whereas overexpression of pancreatic transcription factors in the liver of frogs^26^ and mice^27^ could induce formation of pancreatic cell types. However, post-Yamanaka, researchers could think realistically about deriving β-cells from adult tissue. In terms of technical challenges, it would be easier to generate β-cells from cells that were developmentally more closely related; these would include liver and pancreatic exocrine tissue.^28^ As it turns out during the islet isolation procedure the bulk (98%) of the pancreas is discarded. What if this material could be reprogrammed into β-cells? As each recipient requires up to three islet grafts, this would provide a donor-specific replenishable supply. The ability to perform multiple grafts using reprogrammed cells from a single recipient rather than depending on islet cells from multiple donors would be advantageous from an immune-tolerance stand-point.^13^^,^^29^ Moreover, the reprogrammed cells could be cryopreserved and made available as a recipient-specific top-up supply of islets as the function of the original graft deteriorates over time.

Some progress has been made. When placed in culture, the exocrine material, which is enriched in acinar and ductal cells along with passenger stromal cells and blood vessel-derived cells, attaches to the dish and undergoes a process of dedifferentiation to form a fibroblast-like monolayer that can be repeatedly passaged.^30^^,^^31^ These fibroblasts express surface markers characteristic of mesenchymal stromal cells (MSCs), and in keeping with the properties of MSCs they can be induced to differentiate along adipocyte, osteoblast and chondrocyte lineages. Genetic lineage tracing combined with immunocytochemistry suggests that these MSCs arise in part from amylase- and insulin-expressing cells through a process of epithelial-to-mesenchymal transitioning (EMT).^32^^,^^33^ When cultured in low serum in the presence of growth factors such as glucagon-like peptide 1 (GLP-1) or its analogues, these MSCs can be induced to redifferentiate into cells that express insulin and share some of the properties of β-cells.^34^^,^^35^ These redifferentiated cells have been shown to lower blood glucose levels when engrafted into streptozotocin-diabetic mice,^34^ although the efficiency of the process and the resultant levels of insulin are very low.^35^ An alternative approach is to force overexpression of pancreatic transcription factors such as Pdx1, Ngn3, MafA and Pax4 in combination with growth factors and reagents that are known to modify chromatin structure^33^ (Figure 1B). When this strategy was applied to exocrine-derived MSCs the resulting cells were predominantly α-like, expressing high levels of glucagon but very little insulin. The key to generating a population enriched in β-cells was to use freshly cultured exocrine material and block EMT with rho-associated protein kinase (ROCK) and transforming growth factor β1 (TGFB1) inhibitors. The resultant cells secreted insulin in response to changes in glucose levels in the physiological range and reversed hyperglycaemia when grafted into immune-deficient streptozotocin-induced diabetic mice.^33^

## Conclusion

Considerable progress has been made in deriving β-like cells from pluripotent stem cells or through reprogramming of adult pancreatic exocrine tissue. Islet progenitors can be reproducibly generated from human ESCs and iPSCs and it is envisaged that these progenitors may have clinical utility, and that a bank of HLA-typed iPSCs would be used as a source of material. The differentiated progenitors would be encapsulated and grafted subcutaneously into patients. The expectation is that following engraftment they would mature into functional β-cells.

Transcription factor-mediated reprogramming of exocrine tissue is viewed at present as a patient-specific procedure. This approach has the advantage that the cells would be processed under clinical GMP-grade facilities adjacent to the islet isolation facility. With efficient protocols, there would be no requirement for large scale expansion of the cells as the exocrine tissue would provide adequate material for subsequent transplants to the original recipient. However, if some of the technical hurdles could be overcome, then the MSCs generated from the exocrine tissue, or indeed endocrine tissue, could be expanded several hundred thousand-fold, and subsequently redifferentiated to provide HLA-typed banks of cells for allogeneic transplantation.^36^

Finally, it is worth noting that the default pathway when differentiating pluripotent cells or reprogramming adult exocrine cells is towards the formation of glucagon-secreting α-cells.^33^^,^^37^ It is well established that glucagon secretion in T1D is impaired, and there is further strong evidence from closed loop delivery systems that bi-hormonal delivery of insulin and glucagon has been shown to reduce the risk of hypoglycaemia when compared with delivery of insulin alone.^38^ From a cell therapy perspective, it may be less challenging to make the two cell population separately and combine these to generate α- and β-cell enriched islet-like structures than attempt to generate a functional islet *ab initio*.

From a translational perspective, transplantation of insulin-producing cells is only likely to become a widely adopted therapeutic strategy with significant advances in circumventing immune rejection of the transplanted cells. At present, islet transplantation is only offered to those where the benefits of treatment, i.e. prevention of disabling hypoglycaemia, outweigh the risks of lifelong immunosuppression. Even if donor material was unlimited, the safest way of managing T1D would remain exogenous insulin therapy for the majority. Immunomodulation and immunoisolation are two ways whereby transplanted cells could be protected from immune rejection by the host. Immunomodulation, which involves altering the host immune response, could come in the form of modified regimens using available agents or from more novel strategies.^39^ Immunoisolation would allow transplanted cells to obtain adequate nutrients and secrete insulin in a glucose-dependent manner whilst preventing their allo- or auto-immune rejection. Perhaps, the best example of success is described in a recent study^40^ where a patient was treated with encapsulated islets maintained within an oxygenated chamber. The implanted cells remained glucose responsive at 10 months despite no immunosuppression.

With these advances in generation of insulin-producing cells *in vitro* and parallel progress in prevention in immune rejection, we are now closer than ever towards providing a cell-based solution to patients with T1D.
